# Characterization of a Single-Stranded DNA-Binding-Like Protein from *Nanoarchaeum equitans*—A Nucleic Acid Binding Protein with Broad Substrate Specificity

**DOI:** 10.1371/journal.pone.0126563

**Published:** 2015-05-14

**Authors:** Marcin Olszewski, Jan Balsewicz, Marta Nowak, Natalia Maciejewska, Anna Cyranka-Czaja, Beata Zalewska-Piątek, Rafał Piątek, Józef Kur

**Affiliations:** 1 Gdańsk University of Technology, Chemical Faculty, Department of Molecular Biotechnology and Microbiology, 80–233, Gdańsk, Poland; 2 University of Wroclaw, Faculty of Biotechnology, Department of Protein Engineering, 50–138, Wrocław, Poland; Saint Louis University, UNITED STATES

## Abstract

**Background:**

SSB (single-stranded DNA-binding) proteins play an essential role in all living cells and viruses, as they are involved in processes connected with ssDNA metabolism. There has recently been an increasing interest in SSBs, since they can be applied in molecular biology techniques and analytical methods. *Nanoarchaeum equitans*, the only known representative of *Archaea* phylum Nanoarchaeota, is a hyperthermophilic, nanosized, obligatory parasite/symbiont of *Ignicoccus hospitalis*.

**Results:**

This paper reports on the *ssb*-like gene cloning, gene expression and characterization of a novel nucleic acid binding protein from *Nanoarchaeum equitans* archaeon (*Neq*SSB-like protein). This protein consists of 243 amino acid residues and one OB fold per monomer. It is biologically active as a monomer like as SSBs from some viruses. The *Neq*SSB-like protein displays a low sequence similarity to the *Escherichia coli* SSB, namely 10% identity and 29% similarity, and is the most similar to the *Sulfolobus solfataricus* SSB (14% identity and 32% similarity). The *Neq*SSB-like protein binds to ssDNA, although it can also bind mRNA and, surprisingly, various dsDNA forms, with no structure-dependent preferences as evidenced by gel mobility shift assays. The size of the ssDNA binding site, which was estimated using fluorescence spectroscopy, is 7±1 nt. No salt-dependent binding mode transition was observed. *Neq*SSB-like protein probably utilizes a different model for ssDNA binding than the SSB proteins studied so far. This protein is highly thermostable; the half-life of the ssDNA binding activity is 5 min at 100°C and melting temperature (T_m_) is 100.2°C as shown by differential scanning calorimetry (DSC) analysis.

**Conclusion:**

*Neq*SSB-like protein is a novel highly thermostable protein which possesses a unique broad substrate specificity and is able to bind all types of nucleic acids.

## Background

*Nanoarchaeum equitans* is the only known representative of *Archaea* phylum Nanoarchaeota. Isolated from a submarine hot vent near Kolbeinsey island, north of Iceland, *Nanoarchaeum equitans* is a hyperthermophilic, obligate parasite/symbiont of craenarchaeon *Ignicoccus hospitalis*. The growth of the coculture of these microbes occurs between 70 and 98°C and optimally at 90°C, under strict anaerobic conditions. With a diameter of only 400 nm, *Nanoarchaeum equitans* stands out as one of the tiniest known living organisms [1]. Moreover, next to that of *Candidatus* Carsonella ruddii [2], it has the smallest-ever sequenced genome, which is only 490,885 base pairs long. It is also one of the most compact genomes. Computational predictions suggest that approximately 95% of the DNA encodes proteins or stable RNA. *Nanoarchaeum equitans* lacks genes for most vital metabolic pathways, including lipid, cofactor, amino acid and nucleotide biosynthesis. However, contrary to most known organisms with reduced genomes, it has a full set of the enzymes involved in DNA replication, repair and recombination, one of which is a single-stranded DNA binding like protein [3].

Single-stranded DNA binding proteins are vital elements of living cells and are present in all life domains and in viruses. By means of sequence independent interaction with ssDNA, these proteins prevent strand pairing, secondary structure formation and nuclease degradation [4]. In this way, SSB proteins play a part in every process involving ssDNA metabolism, such as, for instance, replication, recombination and repair [5–8]. SSB proteins can be identified by the presence of a highly conserved DNA binding domain known as the OB fold, which is to say, the oligonucleotide/oligosaccharide/oligopeptide binding fold [9], typically consisting of approximately 100 amino acid residues. However, the subunit composition varies over life domains. The bacterial SSBs characterized to date [10–13], with the exception of those from *Deinococcus-Thermus* [14–16], form homotetramers, while most of eukaryotic SSBs, known as replication protein A (RPAs), usually act in solution as heterotrimers [17]. Current knowledge in respect of the archaeal SSB proteins members of Craenarchaeota phylum posits that they resemble the bacterial-type SSBs in domain organization [18–20], whereas Euryarchaeota have eukaryotic-like RPAs [21,22]. At present, no SSB proteins in the other three *Archaea* phyla, namely Korarchaeota, Thaumarchaeota and Nanoarchaeota, have been reported.

To date, four nanoarchaeal proteins have been published, namely reverse gyrase [23], tRNA splicing endonuclease [24], neelaredoxin [25] and family B DNA polymerase [26]. Interestingly, all of them possess unusual features [23–27]. The sequence analysis of the *Nanoarchaeum equitans* SSB protein indicates that it may also fit the trend. The aim of this study was to clone and overexpress a *Nanoarchaeum equitans* Kin-4m *ssb*-like gene in *E*. *coli*, purify the gene product and study the biochemical properties and protein-DNA interactions of *Neq*SSB-like protein.

## Results

### *Neq*SSB-like protein sequence analysis

**Fig 1:**
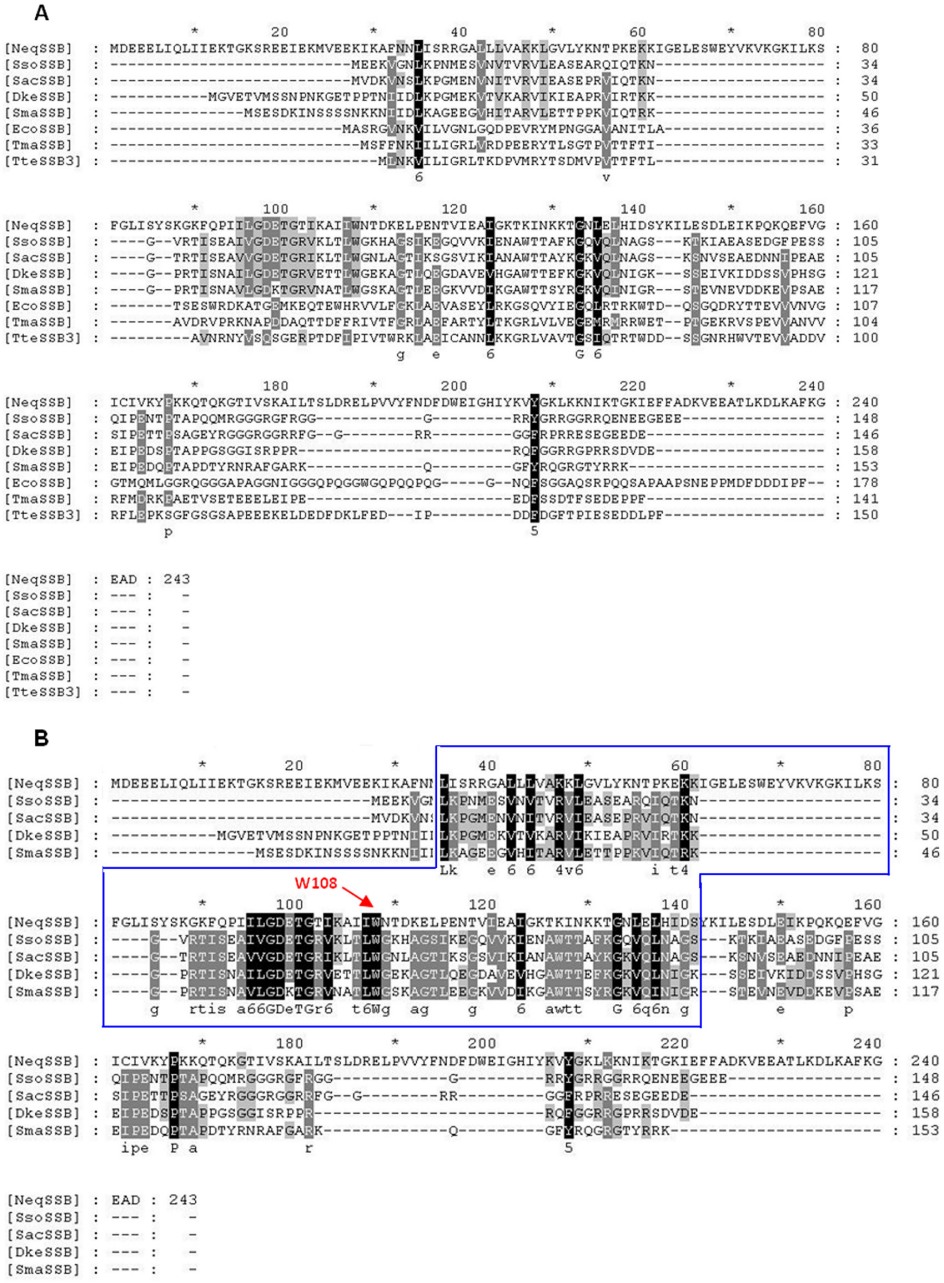
The multiple amino acid alignment. **A** The multiple amino acid alignment of nucleic acid binding protein from *Nanoarchaeum equitans*, bacterial and craenarchaeal SSB proteins. **B** The multiple amino acid alignment of nucleic acid binding protein from *Nanoarchaeum equitans* and craenarchaeal SSB proteins. The alignments were performed by dividing the amino acids into six similarity groups: group 1 V, L, I, M, group 2 W, F, Y, group 3 E, D, group 4 K, R, group 5 Q, D, and group 6 S, T. The capital letters represent single amino acid codes. White fonts on black boxes represent 100% similarity, white fonts on grey boxes denote <80% similarity, and black fonts on grey boxes show <60% similarity. Abbreviations: NeqSSB—nucleic acid binding protein from *Nanoarchaeum equitans* Kin-4M, SsoSSB *Sulfolobus solfataricus* strain P2, SacSSB *Sulfolobus acidocaldarius* DSM 639, SmaSSB *Staphylothermus marinus* F1, DkeSSB *Desulfurococcus kamchatkensis* 1221n, EcoSSB *Escherichia coli* K12, TmaSSB *Thermotoga maritima* strain MSB8, and TteSSB3 *Thermoanerobacter tengcongensis* MB4. The W108 residue, important in base-stacking interactions is indicated in the panel B. The blue box indicates OB-fold region.

The sequence analysis of the *Nanoarchaeum equitans* Kin-4M genome [GenBank: AE017199] indicated the presence of a single *ssb*-like gene. On the basis of this nucleotide sequence, the *Neq*SSB-like protein contains 243 amino acid residues including the N-terminal methionine and has a predicted molecular weight of 27.82 kDa. The RPS-BLAST search revealed one putative DNA binding sequence situated in the region between amino acid residues 71 and 145. Both, this location and the domain length, is unusual, since the OB fold can typically be found near the N-terminus and consists of about 100 amino acid residues. Similarly to *Sso*SSB, *Neq*SSB-like lacks the conserved C-terminal DIPF sequence, although the C-terminal domain does, indeed, have an acidic character. Fig 1A shows the multiple amino acid alignment of the *Nanoarchaeum equitans*, *Sulfolobus solfataricus*, *Sulfolobus acidocaldarius*, *Desulfurococcus kamchatkensis*, *Staphylothermus marinus*, *Escherichia coli*, *Thermotoga maritima* and *Thermoanerobacter tengcongensis* SSB proteins, all of which contain one OB fold domain. Fig 1B shows the multiple amino acid alignment of *Nanoarchaeum equitans* and craenarchaeal SSBs from *Sulfolobus solfataricus*, *Sulfolobus acidocaldarius*, *Desulfurococcus kamchatkensis* and *Staphylothermus marinus*. The identity and similarity between *Neq*SSB-like and *Eco*SSB is 10% and 29% respectively, whereas the alignment between *Neq*SSB-like and *Sso*SSB displays 14% identity and 32% similarity, respectively. These low scores may result from the large difference in the length of amino acid sequences, because sequence of *Neq*SSB-like protein is much longer than the others (from 62 to nearly 100 amino acid residues). The multiple amino acid alignment also indicates that the *Neq*SSB-like W108 residue probably is involved in ssDNA binding, as it is conserved in the *Nanoarchaeum equitans* and craenarchaeal SSB proteins. It corresponds to the base-stacking residue W56 in *Sso*SSB which has already been identified [28].

### The cloning, expression and purification of *Neq*SSB-like

**Fig 2:**
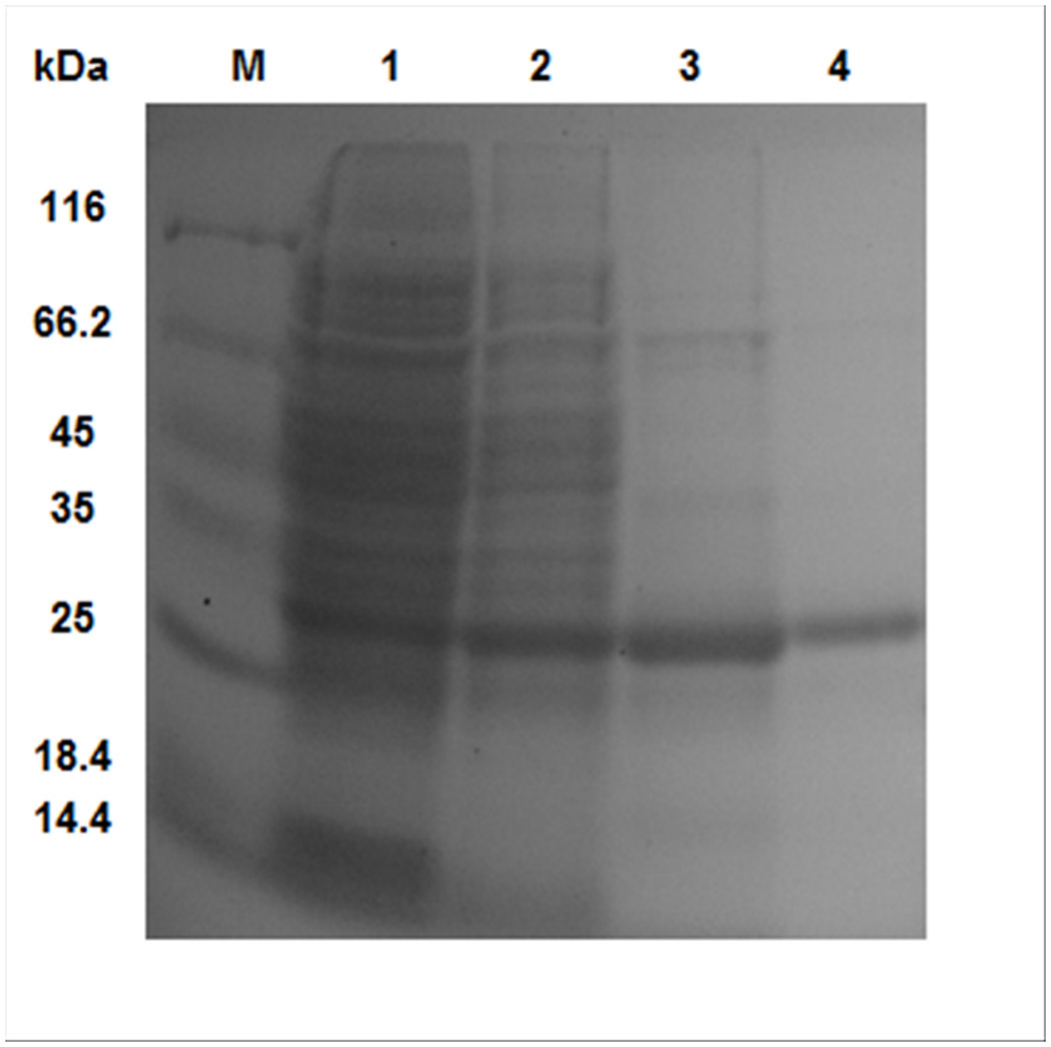
The expression and purification of *Neq*SSB-like from *E*. *coli* TOP10F’+pBAD/NeqSSBHT. The proteins were analyzed on a 12% polyacrylamide gel. Lane M: Unstained Protein Weight Marker (Fermentas, Lithuania), with the molecular mass of proteins marked. Lane 1: soluble protein cell extracts after arabinose induction of protein expression (10 μl). Lane 2: *Neq*SSB-like after the Ni^2+^-affinity chromatography step (10 μl). Lane 3: *Neq*SSB-like after His-tag cleavage with TEV protease (10 μl). Lane 4: *Neq*SSB-like after chromatography on an ssDNA-cellulose column (10 μl).

Using *E*. *coli* TOP10F’ cells carrying the recombinant plasmid pBAD/*Neq*SSB-likeHT, an efficient expression platform under the strict control of a P_BAD_ promoter was constructed. *Neq*SSB-likeHT was produced in a soluble form in cytosol. The SDS-PAGE analysis indicated the presence of a expressed protein with a molecular mass of 28 kDa. The *E*. *coli* overexpression system used in this study allowed 7.5 mg *Neq*SSB-like protein per 1 l of induced culture to be produced. The purity of the protein after all purification steps, namely Ni^2+^-affinity chroamatography, TEV cleavage procedure and ssDNA-cellulose affinity chromatography, was approximately 98%, as evaluated by SDS-PAGE (Fig 2).

### The oligomerization status of *Neq*SSB-like

**Fig 3:**
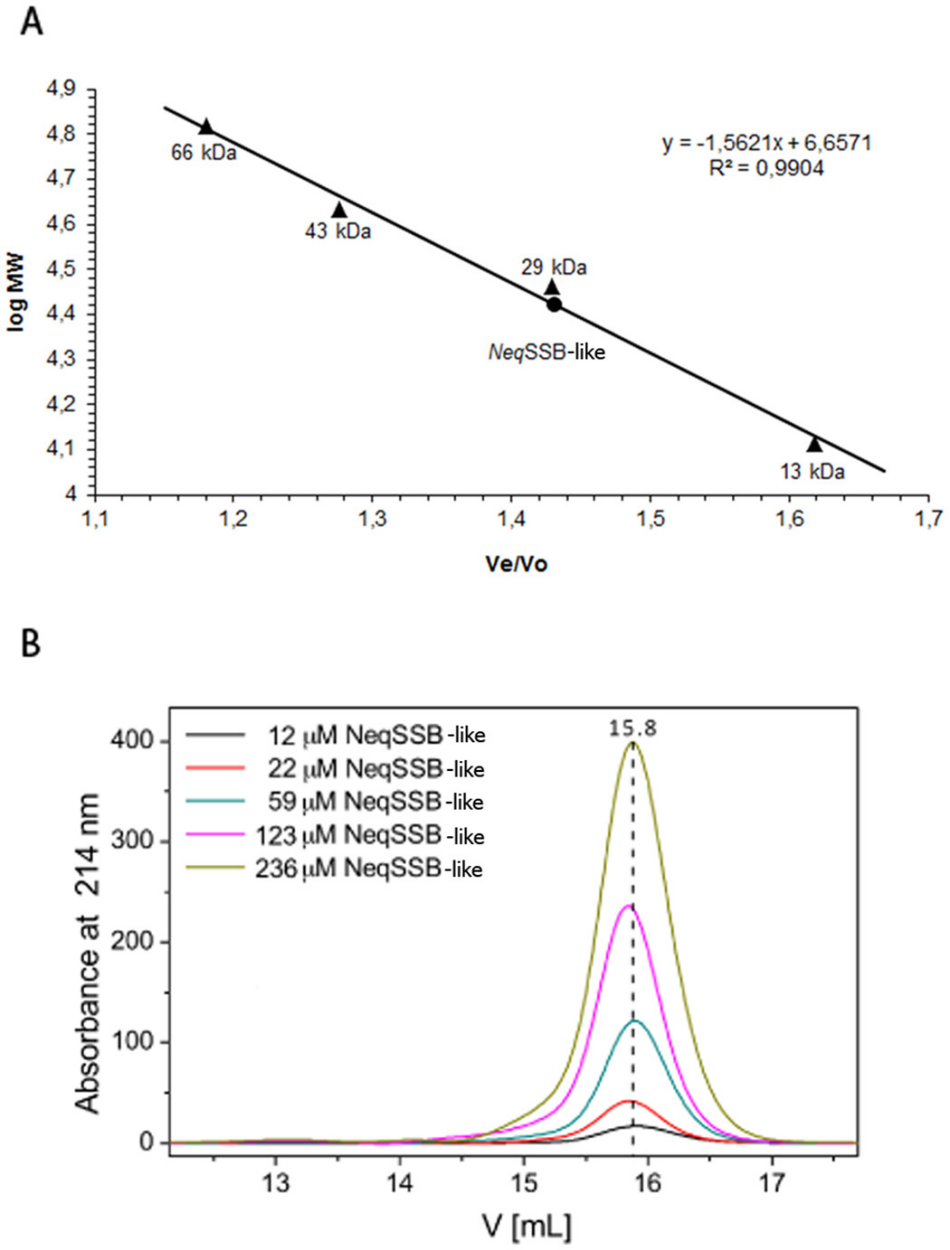
Results of the analytical gel filtration of *Neq*SSB-like on the Superdex 75 10/300 GL column. **A** The standard linear regression curve was generated by plotting the log of the molecular mass of calibration proteins against V_e_/V_0_ value, namely elution volume divided by void volume. The calibration proteins represented by black triangles include bovine albumin (66 kDa), ovalbumin (43 kDa), carbon anhydrase (29 kDa) and ribonuclease A (13 kDa). *Neq*SSB-like is represented by the black circle. The regression curve equation and coefficient of determination are shown. **B** Effects of *Neq*SSB-like protein concentrations on the elution profiles of gel filtration. The entire range of *Neq*SSB-like concentrations (12–236 μM) show an elution volume of 15.8 ml corresponding to the monomeric protein.

The SDS-PAGE analysis of the purified *Neq*SSB-like revealed a single major band with a molecular mass of approximately 28 kDa. The analysis of a purified protein, using analytical gel filtration chromatography at a concentration range between 12 and 236 μM and a different concentration of NaCl (10–300 mM), revealed a homogeneous protein state population (Fig 3). There was one major peak with a molecular mass of 26.36 kDa, as calculated using a regression curve equation (Fig 3). The native molecular mass of the peak represents 0.94 of the monomer mass. Thus *Neq*SSB-like exists in solution as monomer.

**Fig 4:**
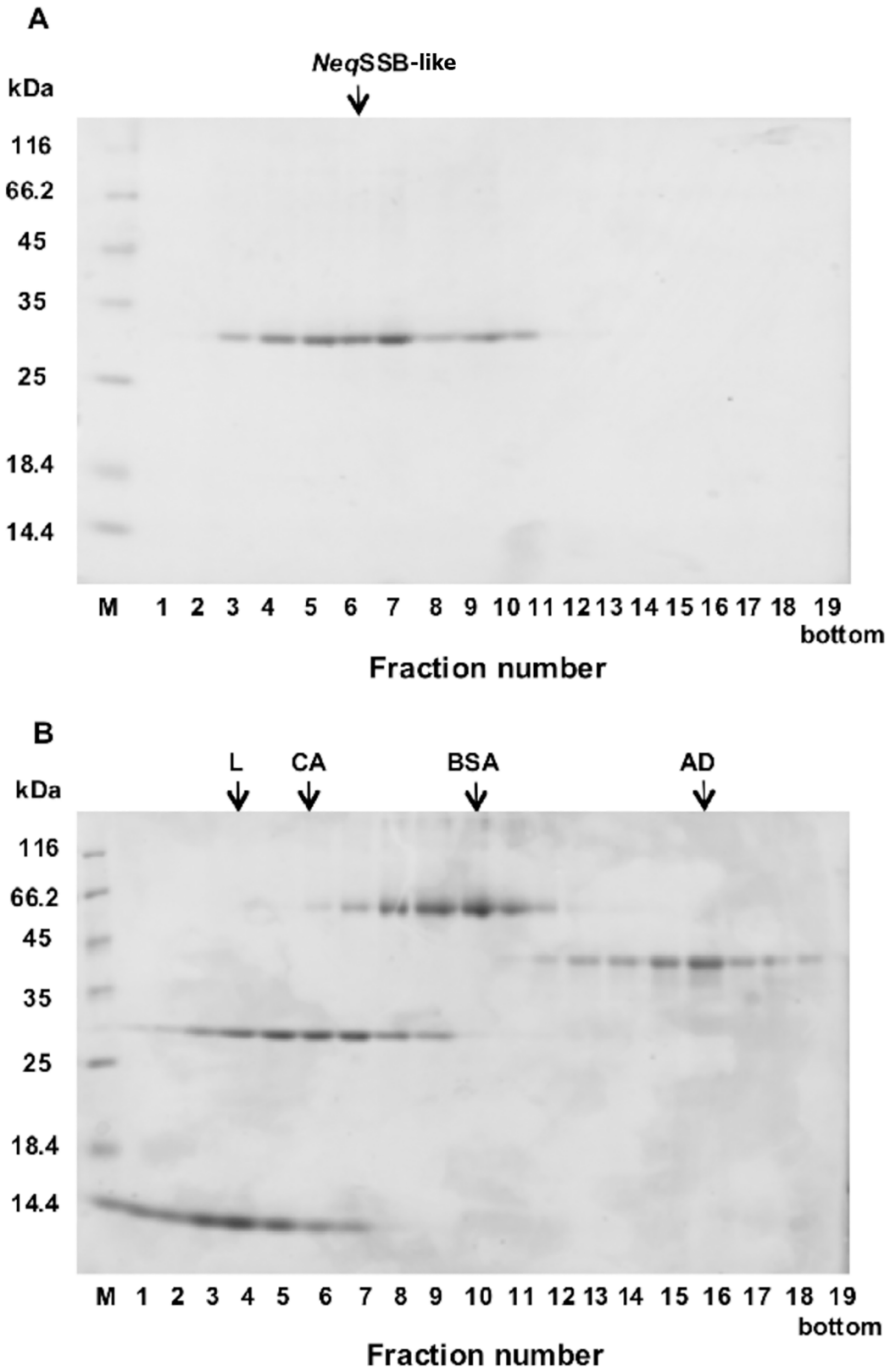
Sedimentation analysis of *Neq*SSB-like (A) and standard proteins (B). The proteins were analyzed on a 12% polyacrylamide gel. Lane M: Unstained Protein Weight Marker (Fermentas, Lithuania), with the molecular mass of proteins marked. Lane 1–19: fraction number. 50 μl of 150 μM *Neq*SSB-like and the corresponding amounts of standard proteins were centrifuged in linear 15 to 30% (w/v) glycerol gradients, as described in the “Methods”. The fractions with proteins were analyzed by SDS-PAGE. The fractions at which the maximal amount of protein appears are shown by arrows in each panel. The standard proteins used are: L, lysozyme (14 kDa); CA, carbonic anhydrase (29 kDa); BSA, bovine serum albumin (66 kDa) and AD, alcohol dehydrogenase (150 kDa). The oligomerization state estimation of *Neq*SSB-like was made with these proteins.

We also used centrifugation in order to analyze the oligomeric state of *Neq*SSB-like in 15 to 30% (w/v) glycerol gradients. To prevent nonspecific aggregation of the protein during the experiments, NaCl at a final concentration of 0.5 M was added to the solutions used for the glycerol gradients. *Neq*SSB-like protein (27.82 kDa) sediments in the position corresponding to a monomeric state (Fig 4). The centrifugation in the glycerol gradients of *Neq*SSB-like was carried out three times and the same sedimentation behaviors were observed in all the independent tests. In addition, the same result was obtained when 50 μl of a 12 μM *Neq*SSB-like protein was examined during this experiment (data not shown), which indicates that the oligomerization state of *Neq*SSB-like is independent of concentration. Thus the sedimentation pattern of *Neq*SSB-like and standard proteins in the glycerol gradients suggests that the *Neq*SSB-like only forms the monomeric structure.

### ssDNA binding properties

**Fig 5:**
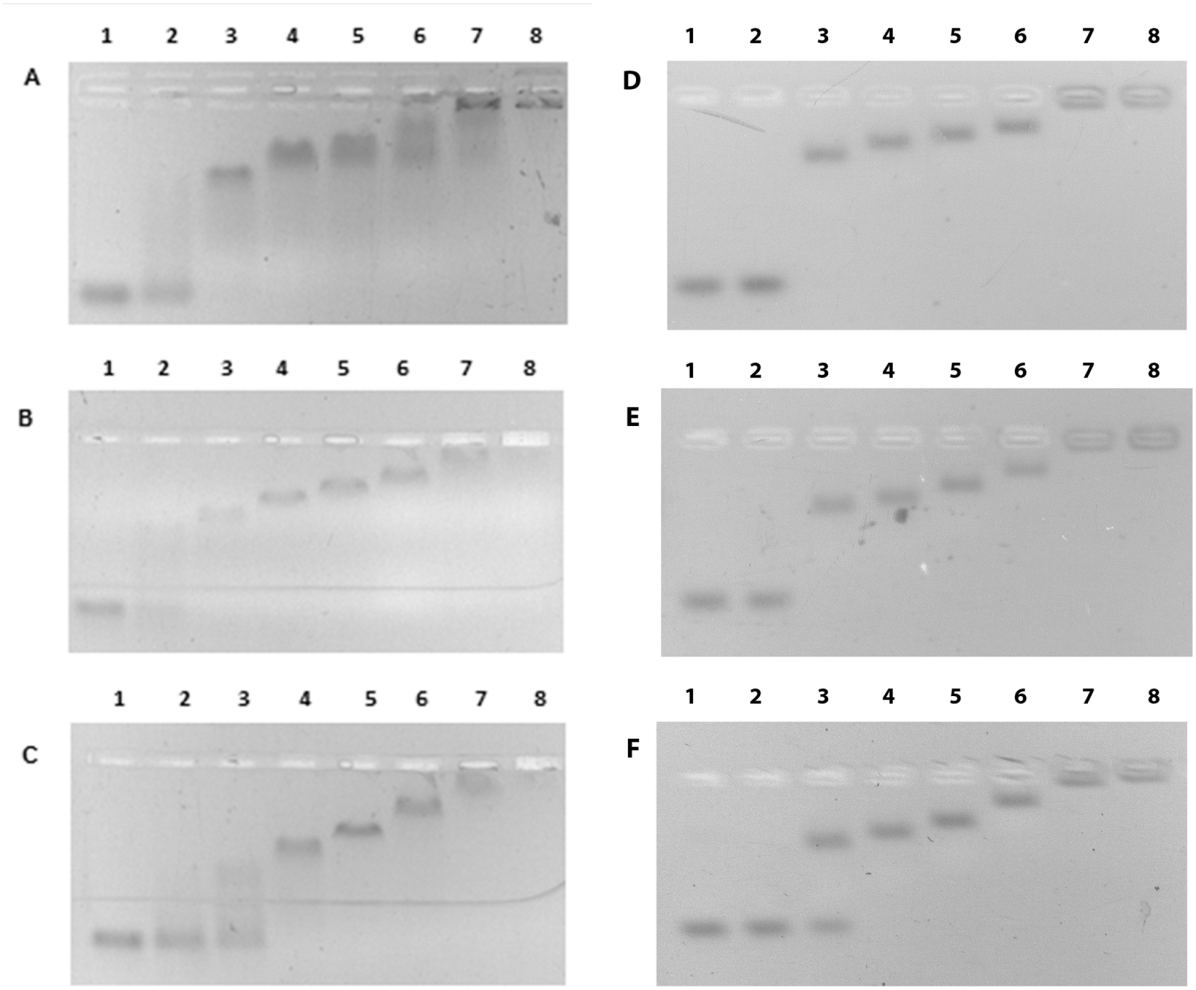
Binding of *Neq*SSB-like to a fixed quantity (10 pmol) of oligonucleotides. **A** (dT)_35_ **B** (dT)_76_ **C** (dT)_120_. Lanes 1–8 contain 0, 10, 20, 40, 80, 160, 320 and 640 pmoles of *Neq*SSB-like, respectively.

Initial studies on ssDNA binding were carried out using a fixed quantity of oligo (dT)_n_ (n = 35, 76, 120). The oligos were incubated with various concentrations of *Neq*SSB-like and the resulting complexes were analyzed, using an agarose gel electrophoresis (Fig 5). The binding reaction started when the *Neq*SSB-like concentration in the test tube was 20 pmol. When an increasing protein amount binds to the oligo (dT)_n_, their mobility in the gel is progressively reduced, resulting in at least five visible complexes, until an end-point, where all available ssDNA stay in the gel well, is reached. These observations do not depend on the length of the oligonucleotide used. In an assay with (dT)_76_ and (dT)_120_, sharp bands were obtained, whereas the picture was somewhat smeary with (dT)_35_. This would suggest that concentration-dependent oligomerization on DNA substrate is occurring.

**Fig 6:**
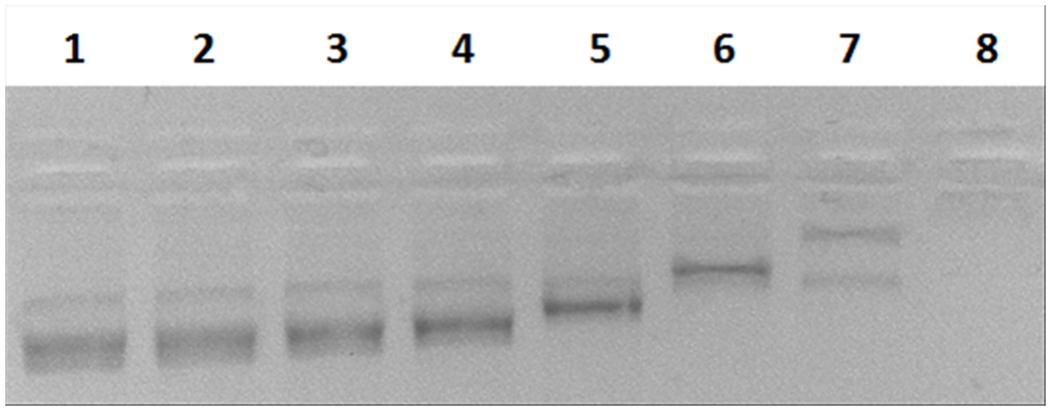
Binding of *Neq*SSB-like to M13 ssDNA. Lanes 1–8 contain (0.07 pmol) of M13 ssDNA and 0, 3.5, 7, 14, 28, 56, 112 and 224 pmoles of *Neq*SSB-like, respectively.

The binding of *Neq*SSB-like to circular M13 ssDNA (6407 bp) was also examined. A fixed quantity of M13 ssDNA was incubated with an increasing amount of *Neq*SSB-like and the resulting complexes were analyzed, using agarose gel electrophoresis. A progressive decrease of M13 ssDNA mobility, along with a quenching of ethidium bromide fluorescence, owing probably to the relaxation of the DNA secondary structures, was observed when increasing the amount of protein added (Fig 6).

**Fig 7:**
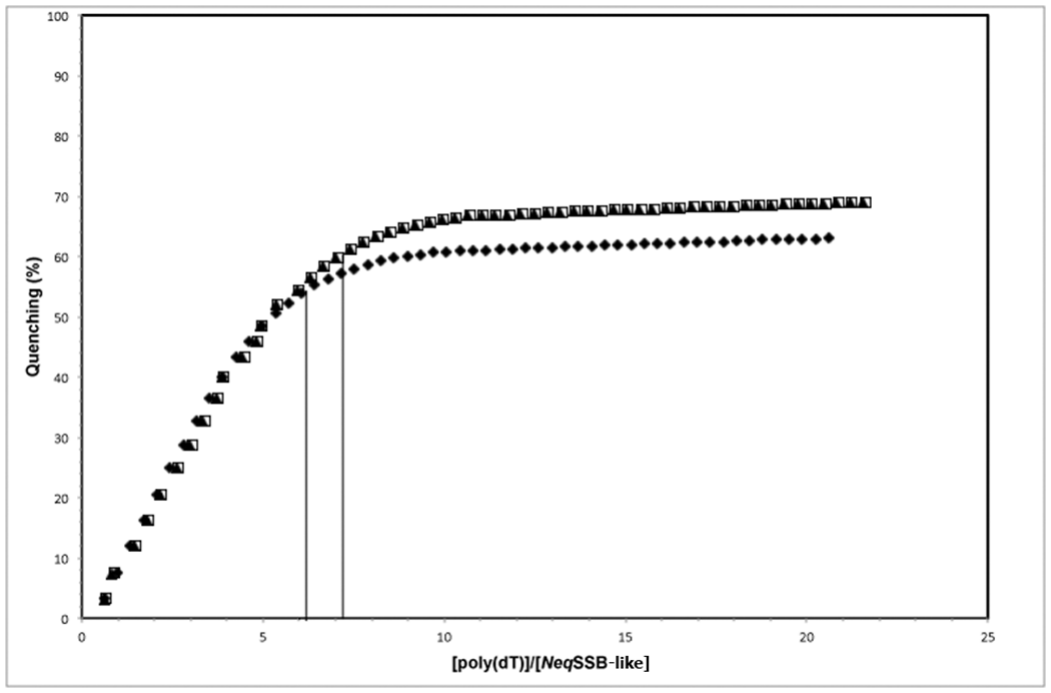
The inverse fluorescence titration of *Neq*SSB-like with poly(dT). 2 nM samples were titrated with a saturating quantity of poly(dT) in 2 mM, represented by the fill diamonds, 100 mM, shown as fill triangles and 500 mM NaCl, given as open squares, in a binding buffer. The vertical lines indicate the binding site size calculated for each assay.

To further explore the ssDNA binding properties of *Neq*SSB-like, fluorescence spectroscopy was used (Fig 7). All the SSBs studied to date show a dramatic decrease in naturally-occurring tryptophan fluorescence when binding to ssDNA. With an excitation wavelength of 295 nm, the emission spectrum of SSB proteins at 25°C had a maximum at 348 nm, consistent with tryptophan. On the addition of a saturating quantity of ssDNA, the intrinsic fluorescence at 348 nm was quenched by 69% in 100 mM and 500 mM NaCl containing buffers and by 63% in a 2 mM NaCl containing buffer. The estimated binding site in the presence of 2, 100 and 500 mM NaCl was determined as 6±1, 7±1 and 7±1 nt, respectively. Therefore, practically no binding mode transition was observed when changing the ionic strength from low to high salt.

Taking into account all the data collected from the *Neq*SSB-like ssDNA binding studies, a cooperative binding model resembling that of DnaA protein complexes is proposed [29]. This would explain the 7 nt binding site and oligonucleotide length-independent number of shifted complexes. The large *Neq*SSB-like complexes bound to ssDNA of various lengths produce differences undistinguishable at the resolution offered by agarose gel electrophoresis. However, the gel smears in the assay with (dT)_35_ may indicate the most cooperative and unorganized binding mode, where only one protein molecule per oligo is bound directly to ssDNA and the others attach to each other cooperatively, forming complexes varying in size. The correctness of this hypothesis is to be further examined by crystallographic studies or high resolution AFM imaging.

### Other nucleic acid binding properties and binding preferences

**Fig 8:**
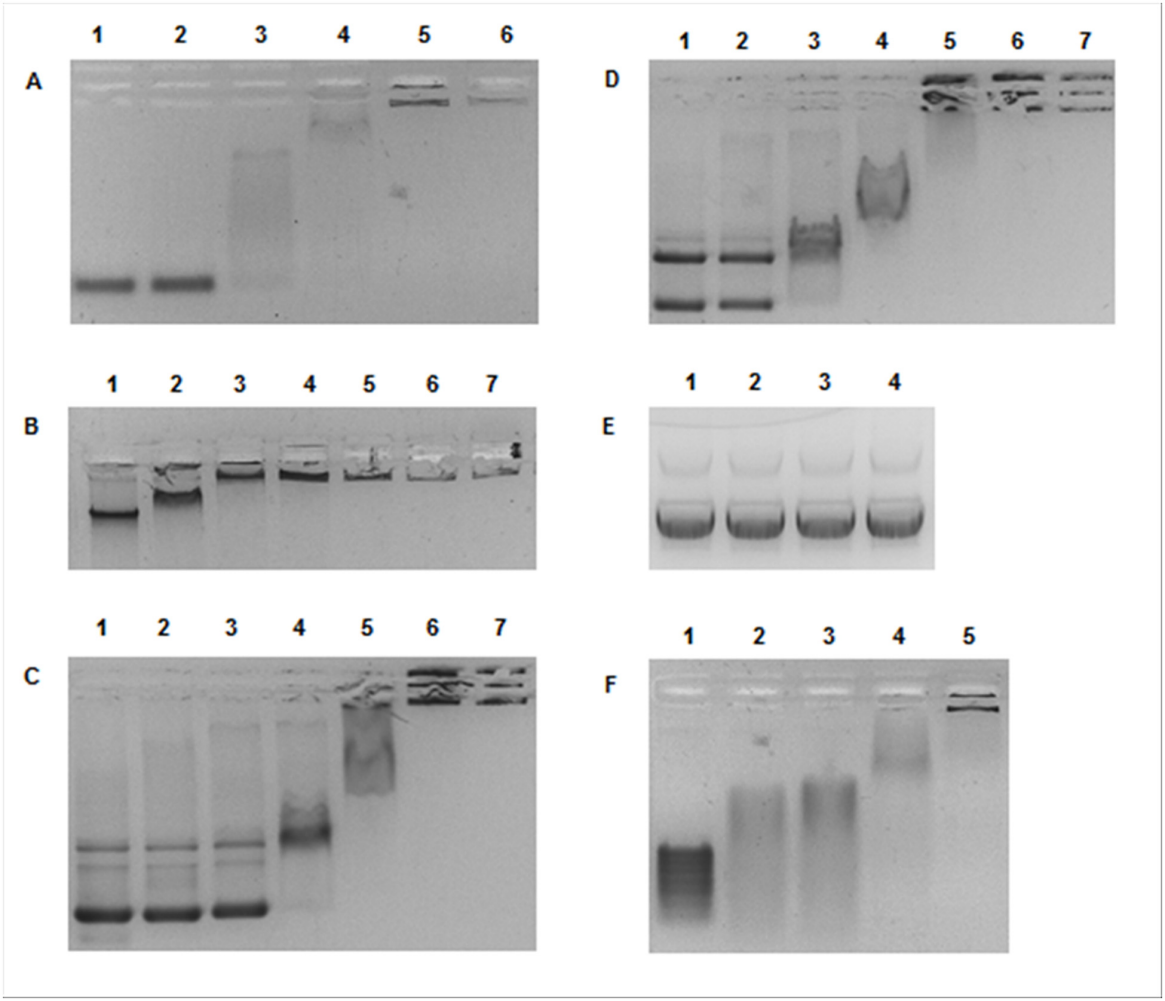
*Neq*SSB-like dsDNA and mRNA binding properties. **A** Binding to 2.5 pmol of 100 bp PCR product. Lanes 1–6 contain 0, 10, 20, 40, 80 and 160 pmoles of *Neq*SSB-like, respectively. **B** Binding to 0.132 pmol of *Escherichia coli* genomic DNA. Lanes 1–7 contain 0, 10, 20, 40, 80, 160 and 320 pmoles of *Neq*SSB-like, respectively. **C** Binding to 0.2 pmol of pDONR201 plasmid DNA (4470 bp). Lanes 1–7 contain 0, 10, 20, 40, 80, 160 and 320 pmoles of *Neq*SSB-like, respectively. **D** Binding to 0.1 pmol of pDONR201 plasmid DNA + 0.05 pmol of linearized pDONR201 plasmid DNA. Lanes 1–7 contain 0, 10, 20, 40, 80, 160 and 320 pmoles of *Neq*SSB-like, respectively. **E** Control binding reaction with 0.2 pmol of pDONR201 plasmid DNA. Lanes 1–4 contain 0, 10, 20 and 40 pmoles of *Taq*SSB, respectively. **F** Binding to 980 ng of mRNA. Lanes 1–5 contain 0, 10, 20, 40, 80 pmoles of *Neq*SSB-like, respectively.

Taking into consideration the low sequence similarity of *Neq*SSB-like to the SSBs studied so far and the extraordinary nature of *Nanoarchaeum equitans*, protein interactions with other nucleic acids were tested. mRNA and dsDNA samples were incubated with increasing amount of *Neq*SSB-like and the resulting complexes were analyzed with agarose gel electrophoresis. The tests showed the capability of mRNA binding, and surprisingly, dsDNA (Fig 8). As with the ssDNA studies, the mobility of mRNA and dsDNA is progressively reduced when increasing the amount of *Neq*SSB-like until the whole sample stays in gel well. No preference for a particular type of dsDNA topology was observed.

**Fig 9:**
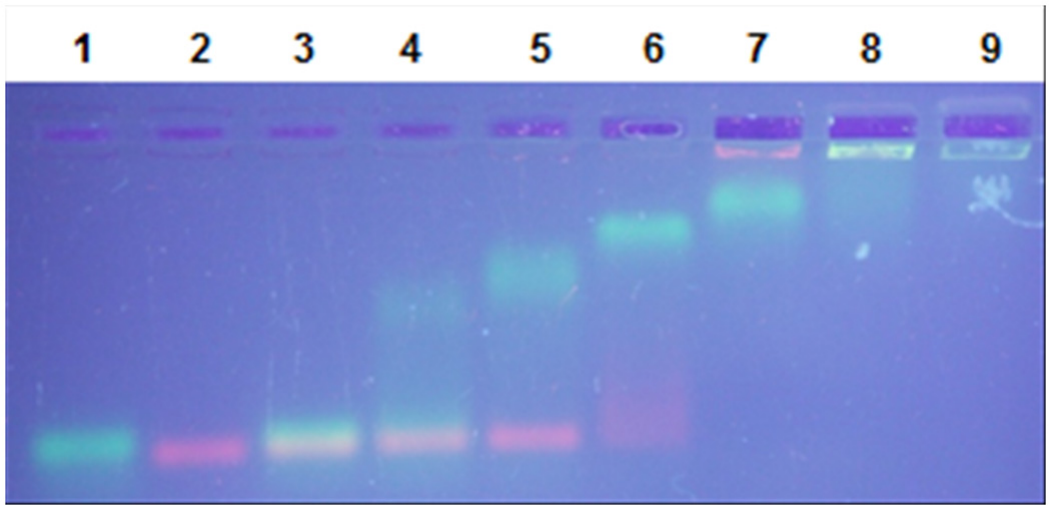
*Neq*SSB-like binding preferences. The reactions contained a fixed quantity of the sample DNA: 10 pmol of (dT)_76_ and 2.5 pmol of 100 bp PCR product. Lane 1: (dT)_76_ with 0 pmol of *Neq*SSB-like. Lane 2: 100 bp with 0 pmol of *Neq*SSB-like. Lane 3: (dT)_76_ and 100 bp PCR product with 0 pmol of *Neq*SSB-like. Lanes 4–9 contain 10, 20, 40, 80, 160 and 320 pmoles of *Neq*SSB-like, respectively.

These experiments gave rise to the question as to whether *Neq*SSB-like can be classified as an SSB protein. To solve this, *Neq*SSB-like was incubated with both ssDNA and dsDNA and the resulting complexes were separated, using agarose gel electrophoresis (Fig 9). The results showed that, with a low protein concentration, ssDNA is bound preferentially and that after saturation, an affinity for dsDNA is observed. Summing up, the results obtained generate speculations about the pleiotropic character of *Neq*SSB-like protein. *Neq*SSB-like may be involved in multiple metabolic pathways with both ds and ssDNA and RNA. Whether this unusual, broad substrate specificity is of ancestral character, or is a consequence of genome reduction remains unknown.

### Kinetic analysis of *Neq*SSB-like interaction with single- and double stranded DNA

**Fig 10:**
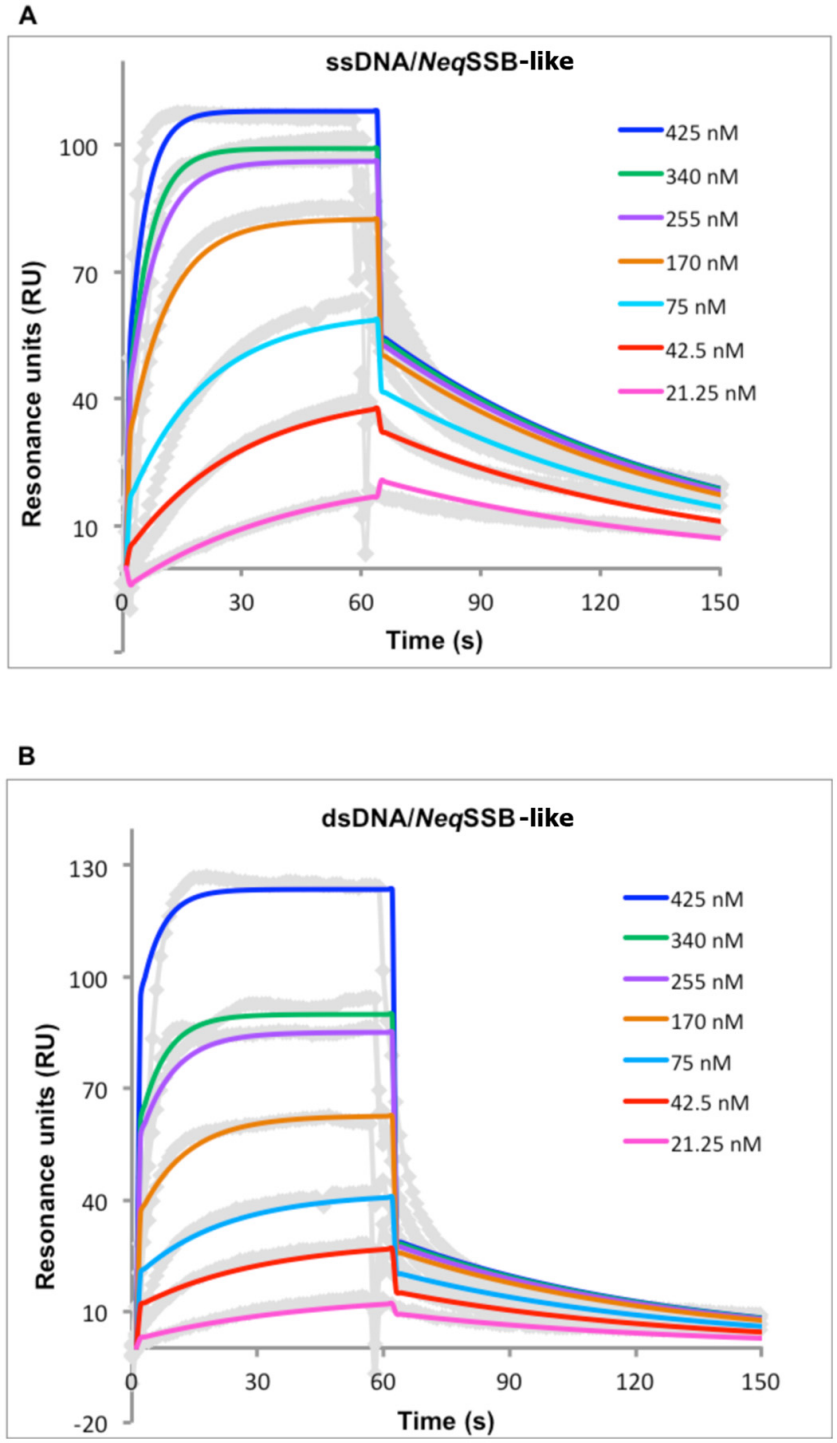
Interaction analysis of *Neq*SSB-like (A) with ssDNA and dsDNA (B). Different concentrations of the protein were injected with a flow rate of 30 μl/min on a streptavidin chip coated with ssDNA 60-mer and dsDNA 60-mer on separate flow channels. A flow cell with streptavidin was used as a reference. After each injection the chip was regenerated with 0.01% SDS. The different colors of the sensograms represent the concentrations of the *Neq*SSB-like injected. Solid lines state for fitted curves. The data were fitted in accordance with the Langmuir model and using BiaEval 3.0 software.

In order to conduct further study on the interaction mode of *Neq*SSB-like with single- and double-stranded DNA (ss- and dsDNA 60-mer), the SPR studies were conceived (Fig 10). They confirmed the unique nature of protein in question, revealing an affinity towards ss- and dsDNA which is unusual for SSB proteins. Using the same ssDNA 60-mer, typical dissociation constants (K_d_) for SSB proteins such as *Eco*SSB or *Mtu*SSB have been determined at the level of nanomolar values [30]. The study also included a control measurement of binding recombinant *Eco*SSB to ssDNA (Table 1). The interaction of *Neq*SSB-like with ssDNA is significantly lower, as is reflected in the K_d_ value of micromolar range (Table 1). Surprisingly, the difference in the preference of *Neq*SSB-like binding to the ssDNA versus the dsDNA has not been meaningfully shown by SPR studies. Though the affinity of *Neq*SSB-like to ssDNA is higher, the values of the kinetic constants are within the same order of magnitude. The dissociation rate for ssDNA is slower, which is reflected in the shape of the sensograms in the dissociation phase and calculated k_off_ values. When compared to other SSB proteins, the main difference in the mode of binding to ssDNA is observed in the dissociation phase; typical values of dissociation rate fall within the 10^–4^ 1/s range [30, 31]. This can easily be explained by the oligomeric state of *Neq*SSB-like, which was determined in this study as monomeric. For other SSB proteins, which are oligomers, most often tetramers, the slow dissociation rate observed by the SPR studies is caused by the avidity effect of multiple binding sites in the oligomer structure.

**Table 1:** Binding parameters for the *Neq*SSB-like interaction with single- and double-stranded DNA.

	K_D_ [M]	k_on_ [1/Ms]	k_off_ [1/s]
*Neq*SSB-like versus ssDNA	2.66 x 10^–6^ ± 0.64	7.91 x 10^3^ ± 1.79	2.39 x 10^–2^ ± 0.46
*Neq*SSB-like versus dsDNA	5.26 x 10^–6^ ± 1.42	8.30 x 10^3^± 2.2	4.89 x 10^–2^ ± 1.7
*Eco*SSB versus ssDNA	5.36 x 10^–9^ ± 1.08	3.45 x 10^4^ ± 0.83	1.85 x 10^–4^ ± 0.23

Binding reactions were performed as described in the “Methods” section. The kinetic data of binding *Neq*SSB-like protein (analyte) to the 60-mer ss- and dsDNA (ligand) were fitted using the Langmuir binding model. The parameters calculated for *Eco*SSB are based on the control experiment conceived within the study. The data shown are the means of at least three independent measurements.

### Thermostability

**Fig 11:**
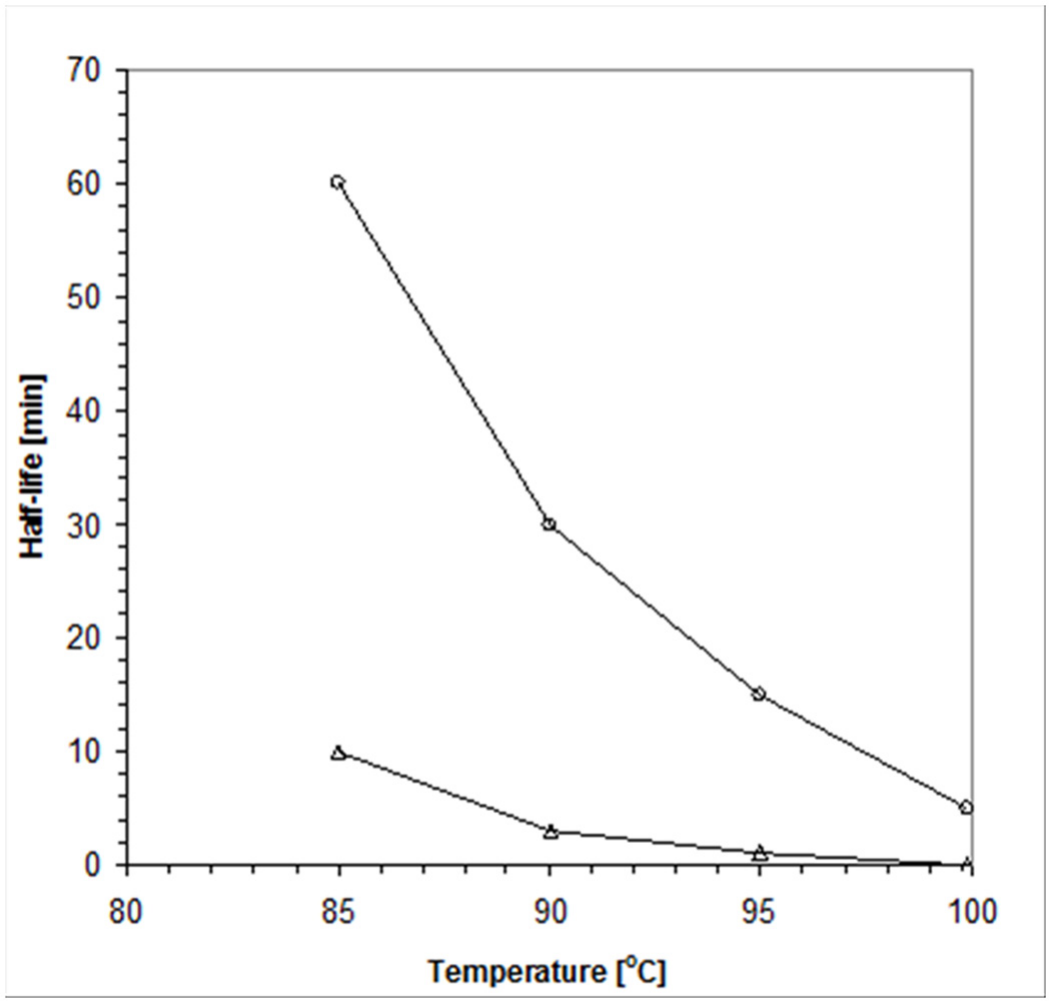
The determination of ssDNA-binding activity half-life, using gel mobility shift assays, for *Neq*SSB-like and *Taq*SSB. *Neq*SSB-like represented by the open circles and *Taq*SSB, shown as open triangles.

The half-lives of *Neq*SSB-like ssDNA-binding activity at different temperatures, as determined by a gel mobility shift assay of (dT)_35_ in 2% agarose gel, are 60 min at 85°C, 30 min at 90°C, 15 min at 95°C and 5 min at 100°C (Fig 11). As regards *Taq*SSB, which was tested as a control protein, the numbers were 10 min at 85°C, 3 min at 90°C, 1 min at 95°C and 0 min at 100°C, as has previously been shown by Dąbrowski *et al*. [14].

**Fig 12:**
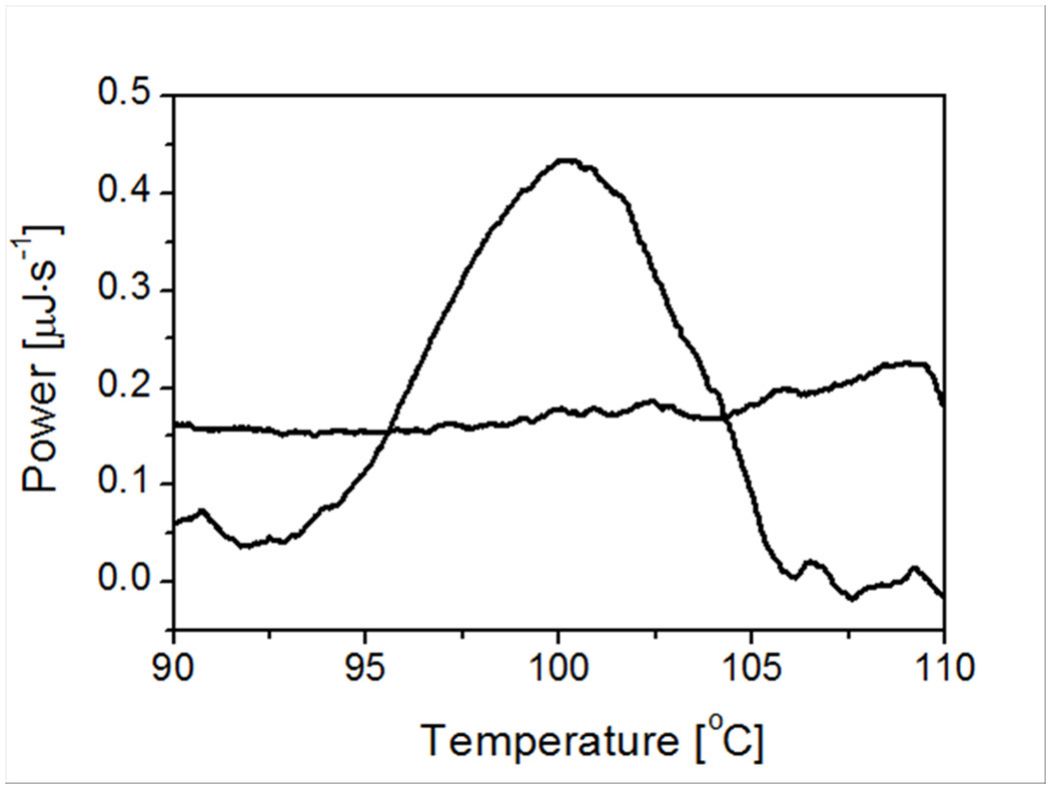
*Neq*SSB-like thermogram. A sample containing 1 mg/ml of purified protein was analyzed in a 20 mM phosphate buffer pH 7.5 containing 150 mM NaCl. *Neq*SSB-like melting temperature is shown.

The Differential Scanning Calorimetry (DSC) analysis of *Neq*SSB-like in Power = f(T) coordinate system showed that thermal unfolding was an irreversible process, as seen on a rescan thermogram (Fig 12). Therefore *Neq*SSB-like does not possess thermodynamic stability. The melting temperature (T_m_) was determined as being 100.2°C, which supports the results obtained for the indirect method. The thermogram analysis indicated no signs of heavy protein aggregation after heat denaturation. Furthermore, the combined results obtained from both methods suggest that the loss of ssDNA binding activity is connected with an irreversible thermal unfolding of *Neq*SSB-like.

### *Neq*SSB-like cannot replace *Eco*SSB *in vivo*

In the complementation experiments, we attempted to replace the resident plasmid (pRPZ146, ori ColEl, Tc^R^, harbouring a wild-type *ssb* gene) from *E*. *coli* RDP268(DE3) (Δ*ssb*::*Kan*) with the plasmids harbouring test *ssb* genes (pET23D(+)NeqSSB-like or pET23-D(+)EcoSSB, ori ColEl, Amp^R^). As SSB is an essential protein, success in replacement of the original Tc^R^ plasmid by the incoming Amp^R^ plasmid, resulting in a Tc^S^, Amp^R^ phenotype, shows that the test SSB complements the Δ*ssb* strain of *E*. *coli*. The pET23D(+)EcoSSB was used as a control to assess the efficacy of the complementation assay. After transformation of modified *E*. *coli* cells using pET23D(+)NeqSSB-like or pET23-D(+)EcoSSB, which encodes resistance against ampicillin, and subsequent inoculations, we could isolate clones that showed resistance to ampicillin and kanamycin but not to tetracycline. These clones must have lost the pRPZ146 plasmid encoding for *Eco*SSB. Additionally, an analysis of plasmid DNA was carried out after plasmid isolation from tested strains. Unfortunately, despite the implementation of a number of passages to replace the resident plasmid pRPZ146 from *E*. *coli* RDP268(DE3) with the plasmids pET23D(+)NeqSSB-like is not achieved.

## Discussion

The research detailed in this work encompassed the cloning, purification and initial characterization of the novel nucleic acid binding protein from the hyperthermophilic archaeon *Nanoarchaeum equitans*. From the sequence analysis obtained, it can be ascertained that *Neq*SSB-like possesses a non-canonical OB fold domain. Using multiple amino acid alignment, the W108 residue was identified as being important in base-stacking interactions. It corresponds to W56 in *Sso*SSB, which has been characterized during thermodynamic studies of ssDNA binding as the greatest DNA binding energy contributor [28].

The gel filtration and sedimentation experiments showed only the monomeric state of *Neq*SSB-like protein that is independent of salt and protein concentration. The observations are the first to date in respect of non-viral SSB proteins. In comparison, the *Sso*SSB protein can form monomers and tetramers in solution [18, 19].

Non-canonical structural properties appear to be important in protein universality and multifunctionality. *Neq*SSB-like possesses the ability to bind all nucleic acid types, with a clear preference for single-stranded forms. On the basis of this study a cooperative model is proposed. Only after solving the *Neq*SSB-like apo and holo crystal structures in order to gain insights into the above-mentioned structural properties will it be possible to present data confirming the suggested binding model.

A salt dependent binding size transition is a common observation in the studies of most of the known SSBs, such as, for example, in *Escherichia coli* SSB [6] and *Deinococcus*-*Thermus*-type SSBs [14–16]. The most extensively studied *Eco*SSB has at least two different DNA binding modes. In high salt concentrations, 65 nucleotides bind per functional protein tetramer, with fluorescence quenching of almost 90%, whereas, in low salt concentrations, 35 nucleotides saturate the protein and quench its intrinsic fluorescence by 53%. The exceptions to this rule were the *Thermotoga maritima* and *Thermotoga neapolitana* SSBs [13]. *Neq*SSB-like also lacks this distinctive property. The spectrofluorimetric studies indicated a salt-independent binding site of approximately 7 nucleotides. In comparison, *Sulfolobus solfataricus* SSB binds 4–5 nt ssDNA per monomer or 20–25 nt per tetramer [18].

The interaction studies by means of SPR assays demonstrated the extraordinary nature of the *Neq*SSB-like in question in respect of both the strength and preference of binding DNA molecules. As the gel-shift assays showed, *Neq*SSB-like can interact with ssDNA, as well as dsDNA, with a slight preference for the first. The values of the kinetic constants are much lower than for the SSB proteins previously studied, though this is also caused by the monomeric structure of the protein, which does not alter the interaction studies by avidity effect, as with other multimeric SSB proteins.

*Neq*SSB-like possesses a fairly high thermostability and its ssDNA binding activity is preserved for 5 minutes, even at a temperature of 100°C. Furthermore, the results obtained from differential scanning microcalorimetry (DSC) demonstrated the melting temperature (T_m_) of *Neq*SSB-like to be 100.2°C. These numbers do not match the SSBs known to be the most thermostatable, namely *Tma*SSB (T_m_ of 109.3C) and *Tne*SSB (T_m_ of 112.5°C) [13], but are significantly higher than those observed for *Taq*SSB (T_m_ of 86.3°C) [14]. *Eco*SSB is essential for the survival of the *E*. *coli* cell [32]. Using an *E*. *coli ssb* mutant strain, we could show that only *Neq*SSB-like can take over the function of *Eco*SSB *in vivo*. We concluded that *Neq*SSB-like is not a single-stranded DNA binding protein involved in routine DNA functions (analogous to the *E*. *coli* SSB protein) or it works incorrectly in the mesophilic *E*. *coli* host because of its about 1000 folds lower activity for ssDNA binding than *Eco*SSB.

## Conclusions

This paper reports on the purification and characterization of the *Nanoarchaeum equitans* SSB protein. Its relation to other known members of this protein class is also presented.

The molecular mass of *Neq*SSB-like, deduced from its 243 amino acid sequence, is 27.82 kDa.

The results of the study show its unique ability to bind different types of nucleic acids including, ssDNA, dsDNA and mRNA, with a confirmed preference to single-stranded substrates. Therefore the *in vivo* multifunctional biological role of *Neq*SSB-like is suggested.

The high thermostability of *Neq*SSB-like, with a half-life of 5 min at 100°C and a DSC-determined T_m_ of 100.2°C, together with its unique binding properties, may offer an attractive tool for thermal nucleic acid amplification techniques.

## Methods

### Cloning of the *ssb*-like gene from *Nanoarchaeum equitans* Kin4-M

*Nanoarchaeum equitans* Kin4-M genome DNA was obtained from the Institute for Microbiology at the University of Regensburg, courtesy of Dr Harald Huber. To confirm the presence of *ssb*-like gene, primers complementary to the flanking sequences, which encode the hypothetical protein (NEQ200) and aspartylo-tRNA (NEQ_t17), were designed and synthesized. The forward primer was 5’ ATATTAAAGCAAACCATTAGACAATTAAAGC (31 nt) and the reverse primer was 5’ TTATTCTCTAAAAGGCTATATAATAGTGG (29 nt). The PCR reaction solution consisted of 0.2 μg of *Nanoarchaeum equitans* Kin4-M genome DNA, 1 μl (10 μM) of each primer, 2.5 μl (10 mM) dNTPs, 2 μl (25 mM) MgCl_2_, 2.5 μl of 10 x Hot Start Buffer (200 mM Tris-HCl pH 8.3, 200 mM KCl, 50 mM (NH_4_)_2_SO_4_), and 2 U of Maxima Hot Start *Taq* DNA Polymerase (Fermentas, Lithuania). 40 cycles were performed, using the Veriti 96 Well Thermal Cycler (Applied Biosystems, USA), with a temperature profile of 60 s at 94°C, 60 s at 56°C and 60 s at 72°C. Specific, approximately 940 bp products were obtained, then purified, using the Agarose-Out DNA Purification Kit (EURx, Poland) and sequenced in order to confirm the presence of the *ssb*-like gene. The respective data are not shown.

Based on the *ssb-*like gene sequence thus confirmed, specific PCR primers were designed and synthesized. The forward primer was NeqSSB-HT-Nco 5’ ggaggaccatggct*caccatcatcatcatcatgagaacctgtacttccagggt***GATGAAGAGGAACTAATACAAC** (75 nt, containing the *Nco*I recognition site, underlined, and the His-Tag domain and TEV cleavage site, given in lower case, italics) and the reverse primer was NeqSSB-HT-Eco 5’ ttagcgaattc**TCAATCGGCCTCTCCTTTAAAAG** (34 nt, containing the *Eco*RI recognition site, underlined). The sections of both primer sequences given in bold are complementary to the nucleotide sequence of the *ssb*-like gene of *Nanoarchaeum equitans* Kin4-M.

The PCR reaction conditions were the same as those already described, with the exception of the temperature profile, which was 60 s at 94°C, 120 s at 51°C and 60 s at 72°C. The PCR products were analyzed, by means of electrophoresis on 2% agarose gel stained with ethidium bromide, at a final concentration of 0.5 μg/ml. Specific, approximately 800 bp products were obtained and isolated from the gel, using the Agarose-Out DNA Purification Kit (EURx, Poland). The PCR product was digested with *Nco*I + *Eco*RI (Fermentas, Lithuania), then purified, using the PCR / DNA Clean-Up Purification Kit (EURx, Poland) and ligated into pBAD/*myc*-His A (Invitrogen, USA) between the *Nco*I and *Eco*RI sites. The *E*. *coli* TOP10F’ cells were transformed with the ligation mixture and 23 colonies were examined for the presence of the *ssb*-like gene from *Nanoarchaeum equitans*, using a gel retardation assay and restriction analysis. One clone was selected and sequenced to confirm the presence of the *ssb*-like gene.

### Protein sequence analysis

The amino acid sequence of *Neq*SSB-like was analyzed using standard protein—protein BLAST and RPS-BLAST. Multiple sequence alignment was generated in ClustalX using PAM 500 score matrix. The results were prepared, using the GeneDoc editor program (copyright Karl Nicholas).

### Expression and purification of *Neq*SSB-like

*E*. *coli* TOP10F’ cells carrying pBAD/*Neq*SSBHT were grown at 37°C in Luria-Bertani medium, supplemented with 100 μg/ml of ampicillin and 12.5 μg/ml of tetracycline to an OD_600_ of 0.4 and were induced by incubation in the presence of arabinose, at a final concentration of 0.02%, for 24 h. The cells were then harvested by centrifugation at 4612xg for 10 min and the pellets were resuspended in 20 ml of buffer A (20 mM Tris-HCl pH 7.9, 0.5 M NaCl, 0.1% Triton X-100 and 5 mM imidazole). The samples were sonicated three times, for 30 s at 4°C, and centrifuged at 10000xg for 15 min. The *Neq*SSB-like was next purified in a three-step procedure. First, the *Neq*SSBHT-like fusion protein was purified, using Ni^2+^-affinity chromatography. The supernatant containing *Neq*SSBHT-like was loaded directly onto an His•Bind Column (Novagen, USA), which has previously been prepared and equilibrated with 30 ml of A buffer. The *Neq*SSBHT-like was eluted with a 5–500 mM imidazole gradient and the elution fraction was then dialyzed against 50 mM Tris-HCl pH 8, 150 mM NaCl and 0.5 mM EDTA. In order to obtain wild-type protein, the His-Tag was then cleaved, using AcTEV Protease (Invitrogen, USA) in accordance with the protocol provided by the supplier. The post-cleavage mixture was dialyzed against the A buffer, loaded onto the prepared and equilibrated column again and flow-through fraction was collected. This fraction containing SSB was first dialyzed against DD buffer (50 mM potassium phosphate buffer pH 7.5, 150 mM NaCl and 1 mM EDTA) and, after rapid dilution to a NaCl concentration of 50 mM, was then loaded onto an ssDNA-cellulose column (5 ml, USB, USA). The *Neq*SSB-like was eluted with 500 mM NaCl and 50% ethylene glycol and the elution fraction was again dialyzed against the DD buffer and concentrated to 1 mg/ml, using the Amicon Ultra-15 Filter Device MWCO 10000 (Millipore, USA). The Ultrafree-MC Centrifugal Filter Devices (Millipore, USA) with microporous membranes and pore sizes of 0.1 μm, were used to remove the particles, precipitates and huge aggregates. The purity of the *Neq*SSB-like was estimated using SDS-PAGE.

### Estimation of the native molecular mass

The molecular mass of *Neq*SSB-like was estimated by means of analytical gel filtration chromatography carried out on a Superdex 75 10/300 GL column (Amersham Biosciences, USA) equilibrated with 20 mM Tris-HCl pH 7.5, 10 mM EDTA and using different concentrations of salt (10, 75, 150 and 300 mM NaCl). The samples containing various concentrations of purified protein (12–236 μM) were eluted with the same buffer, at a flow rate of 0.5 ml/min. The elution profile was monitored by recording the absorbance at 280 nm. The molecular weight of *Neq*SSB-like was determined by comparing the elution pattern with those of standard proteins, namely bovine albumin (66 kDa), ovalbumin (43 kDa), carbon anhydrase (29 kDa) and ribonuclease A (13 kDa). The void column volume was determined by monitoring the elution profile of blue dextran (2000 kDa).

### Sedimentation experiments

Linear 15 to 30% (w/v) glycerol gradients containing loading buffer (50 mM Tris-HCl, pH 7.5, 0.5 M NaCl, 1 mM EDTA and 5 mM β-mercaptoethanol), were prepared in 5 ml Beckman centrifuge tubes. 50 μl of a 12 μM or 150 μM *Neq*SSB-like protein in loading buffer, and the corresponding amounts of standard proteins (lysozyme 14.3 kDa, carbon anhydrase 29 kDa, bovine albumin 66 kDa, alcohol dehydrogenase 150 kDa), were layered over 3.5 ml of the glycerol gradient and were centrifuged in individual tubes. Gradients were centrifuged at 4°C in a Beckman SW 60 rotor at 46,000 rpm for 24 h; fractions were collected from the top. The proteins present in the fractions were separated by SDS-PAGE and after densitometric analysis of the gels, the molecular mass of each protein versus the number of the fraction at which its maximal amount appears was represented.

### Gel mobility shift assays: binding to ssDNA

Studies on ssDNA binding were performed using a fixed quantity (10 pmol) of 5’-end fluorescein-labelled oligonucleotides (dT)_35_, (dT)_76_ and (dT)_120_ or M13 phage ssDNA (0.07 pmol). The oligos were incubated with 10, 20, 40, 80, 160, 320 and 640 pmol of *Neq*SSB-like and the M13 ssDNA was incubated with 3.5, 7, 14, 28, 56, 112 and 224 pmol of *Neq*SSB-like for 10 minutes at 25°C in a binding buffer (20 mM Tris-HCl pH 7.5, 100 mM NaCl and 1 mM EDTA) to a final reaction volume of 20 μl. Subsequently the reaction products with oligos and the reaction products with M13 ssDNA were loaded onto 2% agarose gel, without ethidium bromide and with ethidium bromide, at a final concentration of 0,5 μg/ml, respectively and separated by electrophoresis in a TAE buffer (40 mM Tris acetate pH 7.5 and 1 mM EDTA). The bands corresponding to the unbound ssDNA and various *Neq*SSB-like-ssDNA complexes were visualized under UV light and photographed.

### Gel mobility shift assays: binding to dsDNA

Tests on *Neq*SSB-like dsDNA binding properties were carried out with various DNA types. Specific 100 bp PCR product was obtained using the forward primer M13/pUCf 5’ CCCAGTCACGACGTTGTAAAACG (23 nt) and the reverse primer pUC100r 5’ catgattacgccaagcttgcatgc (24 nt). The PCR reaction solution consisted of 0.1 μg pUC19 DNA (Invitrogen, USA), 4 μl (10 μM) of each primer, 2.5 μl (10 mM) dNTPs, 2 μl (25 mM) MgCl_2_ and 2.5 μl of 10 x Hot Start Buffer (200 mM Tris-HCl pH 8.3, 200 mM KCl and 50 mM (NH_4_)_2_SO_4_), and 2 U of Maxima Hot Start *Taq* DNA Polymerase (Fermentas, Lithuania). 30 cycles were performed, using the Veriti 96 Well Thermal Cycler (Applied Biosystems, USA), with a temperature profile of 60 s at 94°C, 60 s at 57°C and 60 s at 72°C. pDONR201 (Invitrogen, USA) was used as the supercoliled DNA sample, pDONR201, linearized with *Nco*I, as the long, 4470 bp linear DNA sample and *Escherichia coli* genome DNA as the genomic DNA sample. To check the affinity for dsDNA, protein samples were incubated for 10 min at 25°C with fixed quantities of DNA, namely, 2.5 pmol of the 100 bp PCR product with 10, 20, 40, 80 and 160 pmol of *Neq*SSB-like, 0.132 fmol of *Escherichia coli* genome DNA, 0.2 pmol of pDONR201 DNA and mixture of 0.1 pmol pDONR201 plus 0.05 pmol pDONR201/*Nco*I with 10, 20, 40, 80, 160 and 320 pmol of *Neq*SSB-like. A control experiment with 10, 20, 40 pmol of *Taq*SSB protein and 0.2 pmol of pDONR201 DNA was also conducted. All the reaction products were separated, by means of electrophoresis in a TAE buffer, on 1% or 2% agarose gel, stained with ethidium bromide at a final concentration of 0.5 μg/ml, for the plasmid and genomic DNA and the 100 bp of product, respectively. The bands corresponding to unbound DNA and protein-DNA complexes were visualized under UV light and photographed.

### Gel mobility shift assays: binding to mRNA

To verify *Neq*SSB-like mRNA binding properties, 10, 20, 40 and 80 pmol of protein were incubated for 10 minutes at 25°C with 2 μl, corresponding to 980 ng of mRNA, of RiboRuler Low Range RNA Ladder, ready-to-use, 100–1000 bases (Fermentas, Lithuania). Reactions were carried out in a binding buffer (20 mM Tris-HCl pH 7.5, 100 mM NaCl and 1 mM EDTA) to a final volume of 10 μl. The products were then loaded onto 2% agarose gel stained with ethidium bromide, at a final concentration of 0.5 μg/ml, and separated by means of electrophoresis in a TAE buffer. The bands obtained were visualized under UV light and photographed.

### Binding preference studies

Studies into whether *Neq*SSB-like binds preferentially to ssDNA or to dsDNA were carried out using fixed quantities of single stranded oligo (dT)_76_ (10 pmol) and the double stranded 100 bp PCR product (2.5 pmol). Nucleic acid samples were incubated for 10 min at 25°C with 10, 20, 40, 80, 160, and 320 pmol of *Neq*SSB-like in a binding buffer (20 mM Tris-HCl pH 7.5, 100 mM NaCl and 1 mM EDTA) to a final reaction volume of 20 μl. The reaction products were loaded onto 2% agarose gel, stained with ethidium bromide, at a final concentration of 0.5 μg/ml and separated by means of electrophoresis in a TAE buffer. The bands obtained were visualized under UV light and photographed.

### Fluorescence titration

Fluorescence spectroscopy measurements were used to determine the length of binding site and binding properties of *Neq*SSB-like precisely. The fluorescence titrations were made using a Perkin-Elmer LS-5B luminescence spectrometer, as described previously by Curth *et al*. [34]. The binding reactions were assembled in 2 ml of binding buffer (20 mM Tris-HCl pH 7.5 and 1 mM EDTA) containing 2, 100 and 500 mM NaCl and were incubated at 25°C. A constant quantity of *Neq*SSB-like (2 nM) was incubated in the appropriate buffer at 25°C with the amount of poly(dT) (Midland Certified Reagent, Midland, TX) being increased from 0 to 6 nM. The excitation and emission wavelengths were 295 and 348 nm, respectively. The binding curves obtained were analyzed using the model described by Schwarz and Watanabe [35], with *n* as the binding site size, *⍵*·*K* as the cooperative binding affinity and the fluorescence quench *Q*_*f*_ as the parameter. *Q*_*f*_ is defined as (1-F_bound_)/F_free_, where F_bound_ stands for the fluorescence intensity measured for nucleic acid bound protein and F_free_ stands for the fluorescence intensity measured for unbound protein.

### Surface Plasmon Resonance (SPR) measurements

Biacore 3000 was used at a temperature of 25°C for the SPR measurements. The streptavidin coated chip (SA chip, GE Healthcare, USA) was first washed with 10 mM NaOH, 1 M NaCl to remove lose streptavidin. Biotinylated ssDNA 60-mer (5’-AATTCTGGGTGTGTGGGTGTGTGGGTGTGTGGGTGTGGTCAAGTTGACTACGTATACATCbiotin-3’) and dsDNA 60-mer (ssDNA 60-mer with complementary ssDNA) were bound at a level of 500 RU at two different channels, leaving flow cell number 1 as reference. The dsDNA 60-mer was prepared by hybridization of biotinylated ssDNA 60-mer with complementary ssDNA fragment and further agarose gel extraction in order to remove the unpaired ssDNA fragments). Different concentrations of *Neq*SSB-like were injected on the chip, with a flow rate of 30 μl/min, in a HBS buffer (10 mM Hepes, pH 7.4, 150 mM NaCl and 0.005% P20). The analyses of ssDNA and dsDNA interactions with *Neq*SSB-like were conducted in parallel, injecting the protein on the respective flow cells at the same time. Between injections, the flow cells were regenerated with 0.01% SDS. As a control experiment, the interaction between recombinant *Eco*SSB and ssDNA 60-mer was also studied in line with the protocol described for *Neq*SSB-like. The binding parameters for all settings were calculated with BiaEval 3.0 software.

### Thermostability

The *Neq*SSB-like thermostability was determined using both the direct and indirect methods. For the indirect method, a fixed quantity (10 pmol) of 5’-end fluorescein-labelled oligonucleotide (dT)_35_ was added to 20 pmol of *Neq*SSB-like or *Taq*SSB, the control protein, preincubated at, 85, 90, 95 and 100°C for 1, 3, 5, 10,15, 30 and 60 min in a binding buffer (20 mM Tris-HCl pH 7.5, 100 mM NaCl and 1 mM EDTA) to a final reaction volume of 20 μl. After 10 min incubation at 25°C, the SSB-ssDNA complexes were separated from the unbound DNA by means of agarose gel electrophoresis in a TAE buffer on 2% gel.

For the direct method, Differential Scanning Calorimetry (DSC) was used. The measurements were performed using a NanoDSC microcalorimeter (Calorimetry Science Corporation, USA). The *Neq*SSB-like sample, concentrated to 1 mg/ml in 50 mM of potassium phosphate buffer pH 7.5 and 150 mM NaCl was analyzed, by calorimetric scan, between 15 and 120°C, with a scan rate of 1°C/min. The reversibility of the heat unfolding transition was tested by heating and cooling the same sample with a scan rate of 1°C/min. The results were analyzed with the NanoAnalyze Software V 1.1 (TA Instruments, USA).

### Complementation assay

The *E*. *coli* RDP268(DE3) strain harboring pRPZ146 (ColEl *ori*, Tc^R^) plasmid coding wild-type *Eco*SSB protein [32] was used in complementation assay. In this strain expression of target genes cloned in the pET vectors under the control of the T7 promoter is possible and the chromosomal *ssb* gene is replaced by a kanamycin resistance (Δ*ssb*::*Kan* ^*R*^). The pRPZ146 is essential for the survival of the cells and can be replaced by another plasmid, only if it contains a gene whose product can take over *Eco*SSB function *in vivo* [33]. The *E*. *coli* RDP268(DE3)/pRPZ146 cells transformed with pET23D(+)NeqSSB-like plasmid (data not shown) were grown in the presence of ampicillin (100 μg/ml) and kanamycin (25 μg/ml)—present during all the steps. After six consecutive overnight subculturings in 3 ml of 2YT (1.6% tryptone, 1% yeast extract and 0.5% NaCl with or without 1 mM IPTG) containing the same antibiotics, the colonies were patched on 2YT agar plates containing ampicillin alone (100 μg/ml) and on plates containing both tetracycline (25 μg/ml) and ampicillin (100 μg/ml). At the same time, plasmids were isolated from the cultures to estimate the size of plasmids present inside the cells (parallel to inoculating the plates). An Amp^R^ and a Tc^S^ phenotype shows that the incoming plasmid harbors a gene that complements *Eco*SSB.
